# Conversion from Off to On-Pump Coronary Artery Bypass Grafting. Is it Avoidable?

**DOI:** 10.7759/cureus.6791

**Published:** 2020-01-27

**Authors:** Khuzaima Tariq, Kashif Zia, Ali Mangi, Muneer Amanullah, Pervaiz A Chaudry, Musa Karim

**Affiliations:** 1 Cardiac Surgery, National Institute of Cardiovascular Diseases, Karachi, PAK; 2 Pediatric Cardiac Surgery, National Institute of Cardiovascular Disease, Karachi, PAK; 3 Statistician, National Institute of Cardiovascular Diseases, Karachi, PAK

**Keywords:** off-pump coronary artery bypass (opcab), on-pump coronary artery bypass (oncab), conversion

## Abstract

Background

With the emergence of new technologies to stabilize the heart off-pump coronary artery bypass grafting (OPCAB), there is an increasing trend that is being observed throughout the world. In certain circumstances, OPCAB needs to be converted to on-pump CABG (ONCAB). In this study, we aim to identify certain risk factors mandating conversions and their associated short-term outcomes.

Methods

After approval from the institutional ethical review committee and exemption from informed consent, retrospective data of 100 patients meeting the inclusion criteria who underwent OPCAB operations at our institution from August 2018 to July 2019 were included. Preoperative, intraoperative, and postoperative variables were recorded and compared in conversion and non-conversion groups. This study was conducted at the National Institute of Cardiovascular Diseases, Karachi, Pakistan.

Results

A total of 100 patients were included in this study, out of which 82% (82) were male, with age ranging between 18 and 77 years with a mean age of 56.34 ± 8.3 years. In nine of the cases, OPCAB was emergently converted to ONCAB due to arrhythmias. In nine (9%) cases, off-pump CABG was emergently converted to on-pump CABG (ONCAB). Emergent conversion was due to arrhythmias in five cases, due to hypotension during OM graft in two cases, and due to hypotension during ramus graft for the remaining two cases. The emergent conversion was significantly associated with higher New York Heart Association (NYHA) functional classification and comorbid conditions such as chronic obstructive pulmonary disease (COPD).

Conclusion

Emergency conversion from off-pump to OPCAB is the most catastrophic event causing higher morbidity and mortality. Conversion rate was observed to be 9% with arrhythmias being the common cause and patients with higher NYHA status and COPD at baseline were found to be at increased risk of emergency conversion. Considering our results in patients with diagnosed COPD and higher NYHA status, the decision for off-pump CABG should be wisely taken carefully weighing the risks and benefits.

## Introduction

Off-pump coronary artery bypass grafting (OPCAB) is a revascularization modality considered for a selected number of patients in whom postoperative systemic effects of cardiopulmonary bypass machines are recognized as deleterious. The Society of Thoracic Surgeons (STS) data of 118140 CABG-only procedures have reported that OPCABG is associated with decreased mortality and morbidity, including cerebrovascular events, prolonged use of ventilator, deep sternal wound infection, acute renal failure, and reoperation for bleeding [1]. However, in a subset of patients who are initially planned for OPCAB, intraoperative conversion to on-pump coronary artery bypass grafting (ONCAB) is required for completion of the operation, greatly impacting outcomes of converted patients.

The reported incidence of conversion to cardiopulmonary bypass ranges from as low as 1.1% to as high as 16.3% in the available literature. Li et al. have analyzed that OPCAB with intraoperative conversion was associated with a higher proportion of readmissions due to postoperative infection, 19.1% for converted patients compared to 11.9% of readmissions for conventional CABG with cardiopulmonary bypass (CPB) [2]. The common associations with conversions were left main coronary artery disease, heart failure, and three-vessel coronary disease. Patients who require conversion for completion of their surgery may experience a higher rate of morbidity or mortality [3-4].

Numerous clinical scenarios influence the decision to convert patients at the intraoperative stage from an off-pump to an OPCABG procedure, ranging from elective (e.g., need for better surgical exposure, intramyocardial left anterior descending artery (LAD), difficult lateral wall targets) to emergent (e.g., hemodynamic instability, intractable arrhythmias) conversions. It is vital to take a timely decision to improve outcomes.

Common reasons mandating the conversion from an Off-Pump to an On-Pump procedure include the following: hemodynamic instability, failure to adequately expose the target vessel, and global ventricular ischemia as described by Tsaousi et al. in their study [5]. Related articles have reported more or less similar predictors of intraoperative conversions, with most predictors reflecting the higher patient risk for hemodynamic instability or need for multiple bypass grafts [6]. In a study performed by Lim et al., preoperative acute myocardial infarction (AMI) was identified as an independent risk factor for conversion with a p-value of 0.025 [7]. The increased mitral regurgitation (MR) due to a distorted heart, when elevated using heart positioners, has also been observed to be a risk factor for the successful completion of OPCAB. In this study, we aim to determine the incidence of conversions from OPCAB to ONCAB with an evaluation of risk factors causing these conversions and associated outcomes at our institution.

## Materials and methods

After approval from the institutional ethical review committee and exemption from informed consent, retrospective data of 100 patients meeting the inclusion criteria who underwent OPCAB operations at our institution from August 2018 to July 2019 were included.

All patients, including both genders up to the age of 80 years with coronary artery disease undergoing planned OPCAB operations were included in this study. All patients converted to ONCAB due to any reason including hypotension, arrhythmias (e.g. ventricular fibrillation, tachycardia), bleeding, increasing MR, global ischemia, left ventricular (LV) dysfunction with new segmental wall motion abnormality on transesophageal echocardiography, cardiac arrest, difficult targets, and intramyocardial LAD were included. Patients undergoing concomitant CABG and valvular surgery or with a history of previous CABG or any open-heart surgery were excluded from the study.

Preoperative variables like age, gender, New York Heart Association (NYHA) functional classification, previous MI, preoperative ejection fraction (EF %), presence of mild to moderate MR, atrial fibrillation (arrhythmias), number of diseased vessels, left main (LM) disease, and comorbidities like hypertension, diabetes mellitus, dyslipidemia, chronic obstructive pulmonary disease (COPD), renal insufficiency of all the patients in conversion and non-conversion group were recorded. Status of the procedure, either elective or urgent, was also recorded.

Intraoperative variables include the number of grafts, blood transfusions, intra-op MI, arrhythmias, use of intra-aortic balloon pump (IABP), use of cell saver, timing and reason of conversion to cardiopulmonary bypass (CPB) were recorded. Similarly, data on postoperative variables comprised of short-term outcomes including prolonged ventilation, blood transfusions in intensive care unit (ICU), arrhythmias, post-op MI, stroke, reoperation for bleeding/tamponade, use of IABP in ICU, need for postoperative dialysis, length of ICU stay, and operative mortality were obtained.

After anesthesia fitness, OPCAB was performed by experienced cardiac surgeons as well as fellows working at our institution. The anesthesia, surgery, and operative techniques were the same for all of the patients included in this study. Patients were monitored by electrocardiography, pulse oximetry, urine output, invasive arterial and central venous pressure during the procedure.

IBM SPSS, Version 21.0. (IBM Corp., Armonk, NY, US) was used for the analysis of data. Baseline demographic, clinical, preoperative, intraoperative characteristics, and postoperative in-hospital outcomes of emergent conversion group were compared with the non-conversion group by applying appropriate chi-square and Mann-Whitney U test appropriately. Univariate and multivariate logistic regression analysis was performed to determine the predictors of emergent conversion from OPCAB to ONCAB. P-value ≤ 0.05 was statistically significant.

## Results

A total of 100 patients who had undergone OPCAB surgery were included in this study, out of which 82% were male, with age ranging between 18 and 77 years age and a mean of 56.34 ± 8.3 years. Hypertension (66%) was the most frequently observed comorbid condition followed by diabetes (54%) and five (5%) patients had chronic obstructive pulmonary disease (COPD). In nine (9%) cases, OPCAB was emergently converted to ONCAB. The emergent conversion was due to arrhythmias in five cases, due to hypotension during obtuse marginal branch (OM) graft in two cases, and due to hypotension during ramus graft for the remaining two cases. Conversion to ONCAB was found to be associated with higher NYHA functional classification and comorbid conditions such as COPD. Baseline clinical and demographic characteristics stratified by conversion status are presented in Table 1.

**Table 1:** Baseline demographic and clinical characteristics BMI= Body Mass Index, NYHA = New York Heart Association Functional Classification, COPD = Chronic Obstructive Pulmonary Disease, MI= Myocardial Infarction, SD = standard deviation, IQR = interquartile range

Characteristics	Total	Conversion to On-pump		p-value
		Emergent conversion	No conversion	
N	100	9	91	-
Gender				
Male	82% (82)	88.9% (8)	81.3% (74)	0.573
Female	18% (18)	11.1% (1)	18.7% (17)	
Age (years)				
Range	77 - 18	65 - 44	77 - 18	0.665
Mean ± SD	56.34 ± 8.3	52.56 ± 7.92	56.71 ± 8.28	
Median [IQR]	55.5 [63 - 50]	55 [57 - 45]	56 [63 - 52]	
Body Mass Index (kg/m^2^)				
Range	40 - 16.61	29.41 - 18.08	40 - 16.61	0.147
Mean ± SD	25.74 ± 3.95	24.97 ± 3.19	25.82 ± 4.02	
Median [IQR]	25.81 [27.89 - 23.52]	25.35 [26.03 - 24.49]	25.91 [27.89 - 23.51]	
NYHA Class				
I	2% (2)	0% (0)	2.2% (2)	0.023
II	65% (65)	22.2% (2)	69.2% (63)	
III	32% (32)	77.8% (7)	27.5% (25)	
IV	1% (1)	0% (0)	1.1% (1)	
Risk Factors				
Hypertension	66% (66)	66.7% (6)	65.9% (60)	0.965
Diabetes mellitus	54% (54)	44.4% (4)	54.9% (50)	0.547
Smoking	38% (38)	66.7% (6)	35.2% (32)	0.063
Dyslipidemia	14% (14)	0% (0)	15.4% (14)	0.204
COPD	5% (5)	22.2% (2)	3.3% (3)	0.013
Prior MI	36% (36)	44.4% (4)	35.2% (32)	0.580

Preoperative EF of the patients ranged from 25 to 65% with mean of 47.86 ± 10.75%. Mild to moderate MR was present in 30% (30) of the patients and LM disease was observed in 28% (28) of the patients. IABP was used in 23% (23) of the patients and cell saver was used in 13% (13) of the patients. Intraoperative characteristics such as intraoperative MI and arrhythmias were found to be associated with emergent conversion to ONCAB. Pre and intraoperative characteristics stratified by conversion status are presented in Table 2.

**Table 2:** Preoperative and intraoperative characteristics EF: Ejection Fraction; MI: Myocardial Infarction; SD: Standard Deviation; IQR: Interquartile Range; NYHA: New York Heart Association Functional Classification; COPD: Chronic Obstructive Pulmonary Disease; LM: Left Main

Characteristics	Total	Conversion to On-pump		p-value
		Emergent conversion	No conversion	
N	100	9	91	-
Pre-operative EF (%)				
Range	65 - 25	60 - 30	65 - 25	0.063
Mean ± SD	47.86 ± 10.75	41.11 ± 12.44	48.53 ± 10.41	
Median [IQR]	50 [55 - 40]	35 [50 - 30]	50 [55 - 40]	
Left ventricular EF				
<30%	1% (1)	0% (0)	1.1% (1)	0.028
30 to 39%	21% (21)	55.6% (5)	17.6% (16)	
40 to 65%	78% (78)	44.4% (4)	81.3% (74)	
Mitral regurgitation (MR)	30% (30)	33.3% (3)	29.7% (27)	0.417
Mild MR	27% (27)	22.2% (2)	27.5% (25)	0.322
Moderate MR	3% (3)	11.1% (1)	2.2% (2)	
Atrial fibrillation	2% (2)	0% (0)	2.2% (2)	0.653
Triple vessels diseased	95% (95)	100% (9)	94.5% (86)	0.471
LM diseased	28% (28)	11.1% (1)	29.7% (27)	0.237
Procedure status				
Elective	90% (90)	100% (9)	89% (81)	0.295
Urgent	10% (10)	0% (0)	11% (10)	
Number of grafts				
1	1% (1)	0% (0)	1.1% (1)	0.134
2	16% (16)	11.1% (1)	16.5% (15)	
3	61% (61)	33.3% (3)	63.7% (58)	
4	19% (19)	44.4% (4)	16.5% (15)	
5	3% (3)	11.1% (1)	2.2% (2)	
Intraoperative MI	6% (6)	55.6% (5)	1.1% (1)	<0.001
Arrhythmias	5% (5)	55.6% (5)	0% (0)	<0.001
Left ventricular EF (mean ± SD %) by sub-groups of patients				
NYHA class III-IV	46.85 ± 11.41	44.29 ± 12.39	47.54 ± 11.29	0.476
COPD	51 ± 9.62	45 ± 14.14	55 ± 5	0.400
Arrhythmias	44 ± 10.84	44 ± 10.84	-	-

On univariate analysis, NYHA class III-IV, COPD, and number of grafts were found to be significant predictors of emergent conversion from OPCAB to ONCAB with odds ratios of 8.75 [1.7-44.93], 8.38 [1.2-58.77], and 2.77 [1.06-7.23], respectively. However, on multivariate analysis, the independent predictors of emergent conversion to ONCAB were found to be NYHA class III-IV and COPD with significant adjusted odds ratios. The univariate and multivariate analyses for the determinants of emergent conversion from OPCAB to ONCAB are presented in Table 3.

**Table 3:** Determinants of emergent conversion from OPCAB to ONCAB BMI= Body Mass Index, NYHA = New York Heart Association Functional Classification, COPD = Chronic Obstructive Pulmonary Disease, EF = Ejection Fraction, OR = Odds Ratio, CI = Confidence Interval; OPCAB: Off-Pump Coronary Artery Bypass Grafting; ONCAB: On-Pump Coronary Artery Bypass Grafting; LM: Left Main *significant at 5% level of significance

Characteristics	Univariate		Multivariate	
	OR [95% CI]	p-value	OR [95% CI]	p-value
Age (years)	0.95 [0.88-1.02]	0.161	0.81 [0.66-1.01]	0.059
BMI (kg/m^2^)	0.94 [0.78-1.13]	0.538	0.58 [0.3-1.11]	0.098
Male gender	1.84 [0.22-15.69]	0.578	2.65 [0.04-169.88]	0.646
NYHA class III-IV	8.75 [1.7-44.93]	0.009*	46.38 [1.03-2095.53]	0.048*
Hypertension	1.03 [0.24-4.41]	0.965	2.93 [0.24-35.21]	0.396
Diabetes mellitus	0.66 [0.17-2.6]	0.549	5 [0.17-147.66]	0.352
Smoking	3.69 [0.86-15.74]	0.078	39.14 [0.87-1768.42]	0.059
COPD	8.38 [1.2-58.77]	0.032*	1111.73 [1.03-1194893.46]	0.049*
Preoperative EF (%)	0.94 [0.88-1]	0.058	0.93 [0.81-1.07]	0.306
Mitral regurgitation	1.19 [0.28-5.09]	0.819	0.59 [0.04-8.05]	0.693
LM diseased	3.37 [0.4-28.31]	0.262	0.01 [0-3.01]	0.110
Number of grafts	2.77 [1.06-7.23]	0.037*	4.08 [0.46-36.61]	0.209

The total mortality rate was 5% (five), one patient did not survive the operation while four patients died during their postoperative hospital stay. The operative/in-hospital mortality rate was found to be significantly higher in emergent conversion groups as compared to the non-conversion group, 44.4% vs. 1.1%; p<0.001). The emergent conversion of OPCAB to ONCAB was found to be associated with adverse postsurgical in-hospital outcomes such as prolonged ventilation, postoperative MI, stroke, reopen for bleeding/tamponade, and use of IABP in ICU. Postoperative in-hospital outcomes stratified by conversion status are presented in Table 4.

**Table 4:** Postoperative In-hospital outcomes MI = Myocardial Infarction, SD = Standard Deviation, IQR = Interquartile Range, IABP = Intraaortic Balloon Pump, ICU = Intensive Care Unit *significant at 5% level of significance

Characteristics	Total	Conversion to On-pump		p-value
		Emergent conversion	No conversion	
N	100	9	91	-
Operative/in-hospital mortality	5% (5)	44.4% (4)	1.1% (1)	<0.001*
Operative	20% (1)	25% (1)	0% (0)	<0.001*
In-hospital	80% (4)	75% (3)	100% (1)	
Survived surgery	99	8	91	-
Prolonged ventilation	13.1% (13)	62.5% (5)	8.8% (8)	<0.001*
Blood transfusions in ICU	39.4% (39)	75% (6)	36.3% (33)	0.074
Arrhythmias	7.1% (7)	25% (2)	5.5% (5)	0.061
Postoperative MI	5.1% (5)	25% (2)	3.3% (3)	0.013*
Stroke	2% (2)	12.5% (1)	1.1% (1)	0.041*
Reopen for bleeding/tamponade	5.1% (5)	25% (2)	3.3% (3)	0.013*
Use of IABP in ICU	21.2% (21)	100% (8)	14.3% (13)	<0.001*
Need for postoperative dialysis	0% (0)	0% (0)	0% (0)	-
Length of ICU stay (days)				
Range	216 - 18	144 - 18	216 - 24	0.348
Mean ± SD	69 ± 35.45	83.63 ± 47.42	67.71 ± 34.25	
Median (IQR)	48 [90 - 46]	84 [127.5 - 42]	48 [90 - 46]	

## Discussion

Myocardial revascularization can be achieved by coronary artery bypass grafting either via on-pump arrested heart (ONCAB) or OPCAB. An intermediary option is on-pump beating heart operation [8]. This approach continues to use CPB on a beating heart without using cardioplegia for cardiac arrest ensuring myocardial protection. Even with this strategy, the deleterious effects of CPB cannot be eliminated. In high-risk patients, with LV dysfunction, non-dialysis dependent chronic kidney disease, advanced age, female gender, risk of neurological deficit due to severe atherosclerosis, redo operations, OPCAB have shown comparable benefits [9-10].

OPCAB is performed via median sternotomy. Both pleural cavities are opened and deep pericardial sutures are taken for better elevation and exposure of the heart. Target vessels are marked. Octopus tissue stabilizer is used to stabilize the anastomotic site. Urchin heart positioner is applied to access the lateral wall with "off-apex" position. Intracoronary shunts are utilized to maintain the blood flow in view of myocardial protection and to avoid catching the back wall of the vessel during suturing. We routinely use OPCAB for all patients irrespective of their conditions.

The left internal mammary artery (LIMA) was harvested in all operations. The sequence of grafting for distal anastomosis was first LIMA anastomosed to LAD followed by other left-sided targets then right coronary artery (RCA)/ posterior descending artery (PDA) was grafted.

With increasing ease due to technical feasibility, a large number of surgeons prefer OPCAB to avoid proven morbidity associated with the pump run OPCAB, which is proven to be a safe procedure in which grafting is done on a beating heart without compromising myocardial protection, which is generally the main disadvantage of possibly inadequate cardioplegia delivery in critical coronary artery disease during ONCAB [11-12]. A comparison of superiority of OPCAB to ONCAB in terms of safety and better outcome is still debatable given the controversial results from a recently published trial Veterans Affairs Randomized On/Off Bypass (ROOBY) trial, a multicenter trial of 2,203 patients, reported that OPCAB resulted in poorer one-year composite outcomes (i.e., death, MI, and reoperation) [13-16].

In the context of our study, the conversion rate showed in the ROOBY trial is 12.4%, which according to some critics explicates the poor experience of the surgeons involved in the study [17-18]. Poor experience of surgeons is a recognized factor as highlighted by Edgerton in his study. Mukherjee et al. have found conversions to be elective or emergency whereas Edgerton et al. classified converted patients into elective, urgent, and emergent conditions according to the level of urgency of CPB and according to the timing of conversion; they found higher mortality rates in the urgent/emergent and late conversion groups [18-19].

In this study, the conversion rate was found to be 9% with a mortality rate of 44.4% in the converted group, while the mortality rate was 1% in the non-converted group. All the cases included in this study were performed by surgeons experienced in OPCAB and residents/fellows who are in the final year of their training. The only variable among all listed earlier in the methodology section COPD was found to be associated with conversions. Arrhythmias and hypotension were two conditions observed causing conversions. In a recent analysis of STS Adult Cardiac Surgery Database (ACSD) involving over 196,000 patients reported that the conversion rate was 5.5% of which 50% were elective [20-22].

Prolonged ventilation (62.5% vs. 8.8%), blood transfusions in ICU (75% vs. 36.3%), postoperative MI (25% vs. 3.3%), stroke (12.5% vs. 1.1%), and reopening for bleeding/tamponed (25% vs. 3.3%) showed significant difference between the converted and non-converted group. The use of IABP in the operating room (OR) and ICU was almost 100% in converted patients and 14.3% in non-converted patients and extracorporeal membrane oxygenator (ECMO) was used in only one patient with overall high morbidity and mortality. In our study, patients with higher NYHA status and COPD at baseline were found to be at increased risk of emergency conversion. It has been also observed in past that, emergency conversion of OPCAB to ONCAB is more likely in patients with low EF (<30%) or in congestive heart failure (CHF) and preoperative administration of beta-blocker has preventive effects [23]. Time of conversion also reported to have a role in determining the prognosis, it has been observed that timely conversion before hemodynamic collapse leads to relatively better outcomes [20]. Hence, the intraoperative conversion is associated with significantly higher operative mortality and morbidity, as shown in numerous studies; hence, strong consideration should be made in planning OPCAB in patients at increased risk of conversion [20, 24-26]. Therefore, we need to emphasize to build up a strategy to avoid conversions to the largest possible extent and to minimize their after-effects on patients who end up in conversions. The decision regarding early conduct of bypass considering preoperative parameters, the anatomy of target vessels, left ventricular hypertrophy, and intramyocardial LAD, if timely taken can be beneficial. Elective conversion before hemodynamic compromise has less dire consequences as emergency conversion. However, no conversion goes better than all, this requires surgical proficiency along with favorable preoperative patient factors.

Limitations of the study are retrospective data analysis and single-center experience. However, it provides a basis for future studies with larger sample size and multicenter experience.

## Conclusions

Emergency conversion from OPCAB to ONCAB is the most catastrophic event causing higher morbidity and mortality. Conversion rate was observed to be 9% with arrhythmias being the common cause and patients with higher NYHA status and COPD at baseline were found to be at increased risk of emergency conversion. Considering our results in patients with diagnosed COPD and higher NYHA status, the decision for OPCAB should be wisely taken carefully weighing the risks and benefits.

## References

[1] Cleveland Jr JC, Shroyer AL, Chen AY, Peterson E, Grover FL (2001). Off-pump coronary artery bypass grafting decreases risk-adjusted mortality and morbidity. Ann Thorac Surg.

[2] Li Z, Amsterdam EA, Danielsen B, Hoegh H, Young JN, Armstrong EJ (2014). Intraoperative conversion from off-pump to on-pump coronary artery bypass is associated with increased 30-day hospital readmission. Ann Thorac Surg.

[3] Légaré JF, Buth KJ, Hirsch GM (2005). Conversion to on pump from OPCAB is associated with increased mortality: results from a randomized controlled trial. Eur J Cardiothorac Surg.

[4] Patel NC, Patel NU, Loulmet DF, McCabe JC, Subramanian VA (2004). Emergency conversion to cardiopulmonary bypass during attempted off-pump revascularization results in increased morbidity and mortality. J Thorac Cardiovasc Surg.

[5] Tsaousi G, Pitsis AA, Ioannidis GD, Vasilakos DG (2014). A multidisciplinary approach to unplanned conversion from off-pump to on-pump beating heart coronary artery revascularization in patients with compromised left ventricular function. Crit Care Res Pract.

[6] Jin R, Hiratzka LF, Grunkemeier GL (2005). Krause A, Page III US: Aborted off-pump coronary artery bypass patients have much worse outcomes than on-pump or successful off-pump patients. Circulation.

[7] Lim J, Lee WY, Ra YJ, Jeong JH, Ko HH (2017). Analysis of risk factors for conversion from off-pump to on-pump coronary artery bypass graft. Korean J Thorac Cardiovasc Surg.

[8] Perrault LP, Menasché P, Peynet J (1997). On-pump, beating-heart coronary artery operations in high-risk patients: an acceptable trade-off?. Ann Thorac Surg.

[9] Kowalewski M, Pawliszak W, Malvindi PG (2016). Off-pump coronary artery bypass grafting improves short-term outcomes in high-risk patients compared with on-pump coronary artery bypass grafting: meta-analysis. J Thorac Cardiovasc Surg.

[10] Guida GA, Chivasso P, Fudulu D (2016). Off-pump coronary artery bypass grafting in high-risk patients: a review. J Thorac Dis.

[11] Sergeant P (2016). We should ban the OPCAB approach in CABG, just as we should ban jetliners and bicycles, or maybe not!. J Thorac Dis.

[12] Raja SG (2016). Two decades of off-pump coronary artery bypass surgery: Harefield experience. J Thorac Dis.

[13] Shroyer AL, Grover FL, Hattler B (2009). On-pump versus off-pump coronary-artery bypass surgery. N Engl J Med.

[14] Widimsky P, Straka Z, Stros P (2004). One-year coronary bypass graft patency: a randomized comparison between off-pump and on-pump surgery angiographic results of the PRAGUE-4 trial. Circulation.

[15] Chu D, Bakaeen FG, Dao TK, LeMaire SA, Coselli JS, Huh J (2009). On-pump versus off-pump coronary artery bypass grafting in a cohort of 63,000 patients. Ann Thorac Surg.

[16] Hattler B, Messenger JC, Shroyer AL (2012). Off-Pump coronary artery bypass surgery is associated with worse arterial and saphenous vein graft patency and less effective revascularization: Results from the Veterans Affairs Randomized On/Off Bypass (ROOBY) trial. Circulation.

[17] Guyton RA (2010). Surgery: On-pump or off-pump CABG surgery—under the spotlight. Nat Rev Cardiol.

[18] Mukherjee D, Ahmed K, Baig K, Patel VM, Darzi A, Athanasiou T (2011). Conversion and safety in off-pump coronary artery bypass: a system failure that needs re-emphasis. Ann Thorac Surg.

[19] Edgerton JR, Dewey TM, Magee MJ, Herbert MA, Prince SL, Jones KK, Mack MJ (2003). Conversion in off-pump coronary artery bypass grafting: an analysis of predictors and outcomes. Ann Thorac Surg.

[20] Keeling B, Thourani V, Aliawadi G (2017). Conversion from off-pump coronary artery bypass grafting to on-pump coronary artery bypass grafting. Ann Thorac Surg.

[21] Mukherjee D, Ashrafian H, Kourliouros A, Ahmed K, Darzi A, Athanasiou T (2012). Intra-operative conversion is a cause of masked mortality in off-pump coronary artery bypass: a meta-analysis. Eur J Cardiothorac Surg.

[22] Lamy A, Devereaux PJ, Prabhakaran D (2012). Off-pump or on-pump coronary-artery bypass grafting at 30 days. N Engl J Med.

[23] Yoon SS, Bang JH, Jeong SS, Jeong JH, Woo JS (2017). Risk factors of on-pump conversion during off-pump coronary artery bypass graft. Korean J Thorac Cardiovasc Surg.

[24] van Dijk D, Nierich AP, Jansen EW (2001). Early outcome after off-pump versus on-pump coronary bypass surgery: results from a randomized study. Circulation.

[25] Novitzky D, Baltz JH, Hattler B, Collins JF, Kozora E, Shroyer AL, Grover FL (2011). Outcomes after conversion in the Veterans Affairs randomized on versus off bypass trial. Ann Thorac Surg.

[26] Hassanein M, El-Awady W (2016). Timing of conversion of off-pump CABG to on-pump CABG is the most important factor determining the outcome of the converted patients. J Egypt Soc Cardiovasc Surg.

